# Propolis: A Natural Bioactive Compound with Emerging Roles in Functional Food Applications

**DOI:** 10.3390/ijms27135755

**Published:** 2026-06-25

**Authors:** Mohamed Hussein Hamdy Roby, Mohamed Mahmoud Shaban Hassan, Adel Abdelrazek Abdelazim Mohdaly, Tugba Ozdal

**Affiliations:** 1 Department of Food Science and Technology, Faculty of Agriculture, Fayoum University, Fayoum 63514, Egypt; mmm12@fayoum.edu.eg (M.M.S.H.); aam01@fayoum.edu.eg (A.A.A.M.); 2 Department of Genetics and Bioengineering, Faculty of Engineering and Natural Sciences, Istanbul Okan University, 34959 Tuzla, Istanbul, Türkiye; tugba.ozdal@okan.edu.tr

**Keywords:** bee propolis, functional foods, health benefits, natural food preservative, scavenging activity, bioactive compounds and microencapsulation

## Abstract

Propolis, a resinous substance biosynthesized by honeybees from plant exudates and beeswax, has been valued for centuries in traditional medicine and is now increasingly recognized as a promising natural bioactive compound for functional food applications. Its complex phytochemical profile, mainly comprising flavonoids, phenolic acids, and terpenoids, confers potent antioxidant, antimicrobial, and anti-inflammatory properties that position it as a compelling candidate for use as a natural food preservative and bioactive additive. Despite this considerable potential, the widespread incorporation of propolis into food systems remains largely constrained by two main physicochemical limitations: its intense characteristic aroma, attributable to volatile terpenes and phenolic esters, which adversely affects sensory acceptance, and its inherent hydrophobicity, which prevents uniform dispersion in aqueous food matrices. This review critically examines three major technological strategies developed to overcome these barriers: (i) microencapsulation employing biopolymer wall materials, including alginate, chitosan, whey protein, and arabic gum, to mask organoleptic properties and enable controlled release; (ii) nanoemulsification to enhance water dispersibility and improve oral bioavailability; and (iii) the formulation of water-soluble propolis extracts through polyethylene glycol-based solvents or cyclodextrin complexation. In addition, this review provides a comprehensive assessment of the global chemical diversity of propolis and its bioactive properties as they relate to food preservation efficacy. Notwithstanding recent technological advances, critical research gaps persist regarding optimal effective concentrations, validated delivery systems, and scalable formulation strategies necessary for commercial food-grade applications. Addressing these gaps is essential for propolis to fulfill its considerable potential as a safe, widely accepted, and commercially viable natural food additive in next-generation functional food systems.

## 1. Introduction

Propolis is a resinous hive-derived substance, with a wide range of colors (red, green, brown, and yellow), that honeybees (*Apis mellifera*) collect and process from a diverse array of plant species, including alder, eucalyptus, poplar, birch, and acacia. The collected material is subsequently transported to the hive and combined with beeswax, yielding a highly adhesive substance [1,2,3]. Propolis is collected by all species of Apis, as well as stingless bees, such as Melipona and Trigona [4]. Propolis is a complex resinous material composed of plant-derived resins, beeswax, essential oils, and pollen, which honeybees employ as both a structural sealant and an antimicrobial defense component within the hive [5]. Propolis is broadly classified into two categories—temperate and tropical—each exhibiting distinct chemical profiles.

Over many years, the chemical properties and characteristics of propolis have been intensively explored, with data published in various scientific articles across the world. The chemical composition of propolis is highly variable, depending primarily on the botanical origin, geographic location, climate, harvest season, and bee species [6,7,8,9,10]. Accordingly, a wide range of chemical constituents has been identified and reported in the literature [11,12].

Propolis has attracted considerable scientific attention owing to its documented biological activities, including antiviral, antibacterial, cariostatic, antioxidant, hepatoprotective, and anticancer properties [3,13,14,15,16,17,18]. As a result, growing scientific interest has emerged in leveraging these documented biological activities to improve the nutritional quality, functional value, and overall safety of food products.

The potential benefits of propolis include its potent antimicrobial, anti-inflammatory, and immunomodulatory properties, which offer promising avenues for addressing various health challenges, including the growing threat of antibiotic resistance [19,20,21]. This natural resin, rich in flavonoids, phenolic compounds, and terpenes, exhibits diverse pharmacological activities that could serve as alternative therapies for chronic diseases [22]. Specifically, its comprehensive phytochemical profile contributes to its antioxidant capabilities, mitigating oxidative stress implicated in numerous pathologies, as well as to its documented efficacy against various microbial pathogens [23,24]. This broad-spectrum antimicrobial action, especially against antibiotic-resistant strains, positions propolis as a valuable natural compound for combating emerging infectious diseases, thereby reducing reliance on conventional antibiotics [25,26]. Moreover, the presence of unique phenolic compounds, terpenes, and polysaccharides in propolis from diverse bee species, such as stingless bees, suggests an expanded potential for novel bioactive compounds that warrant further investigation for their therapeutic applications and sustainable utilization [27]. Its ability to scavenge free radicals and bolster antioxidant enzyme activity further highlights its potential for prophylactic and therapeutic interventions against oxidative damage [28]. Furthermore, the anti-inflammatory and antiviral characteristics of propolis underscore its utility in managing conditions ranging from upper respiratory tract infections to more complex chronic inflammatory states [20]. The multifaceted biological activities of propolis, including its antibacterial, antifungal, and antiviral properties, are particularly relevant given the global rise in multidrug-resistant pathogens [26]. These diverse biological effects are primarily attributable to the complex array of phenolic substances, particularly flavonoids [29]. Propolis is effective against a wide range of microbes, including bacteria, viruses, and fungi. This indicates its potential use as a treatment to increase the shelf life of food [30,31,32].

This review is structured as follows: it first presents the primary chemical classes identified across propolis samples worldwide, then analyzes the key drivers of compositional variation, appraises recent advances in analytical methodology, highlights newly discovered or newly characterized bioactive molecules, and finally identifies critical gaps demanding attention. Throughout, priority is given to research published until 2026, enabling an account that accurately reflects the current frontier of the field.

## 2. Physical Characteristics of Propolis

**Figure 1:**
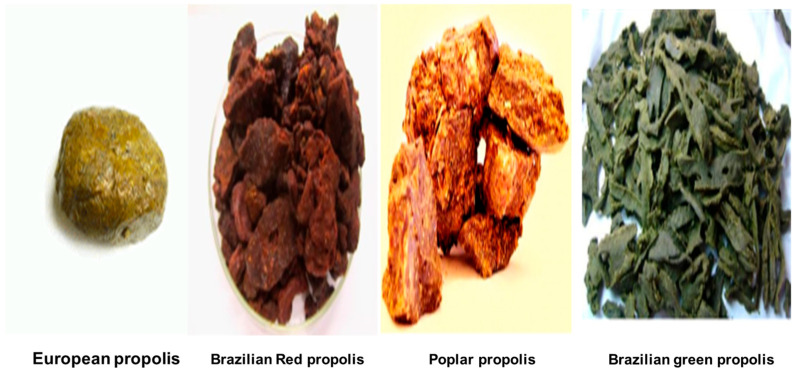
Examples and appearance of different types of propolis. Source: developed by the authors.

As mentioned previously, propolis exhibits a complex and variable chemical composition that significantly influences its physical characteristics. This variability is largely dictated by environmental factors, such as geographical location, prevalent flora, and climatic conditions, leading to distinct physicochemical profiles, even among propolis samples from proximate regions. Consequently, the intrinsic chemical diversity of propolis, arising from its botanical origins and the specific bee species involved in its production, presents substantial challenges in establishing standardized quality control measures and identifying definitive marker compounds [33]. This inherent complexity necessitates comprehensive analytical approaches to characterize propolis and elucidate the intricate relationships between its phytochemical constituents and its observed physical properties [34]. The physical appearance of propolis, encompassing aspects such as color, consistency, and fragrance, is directly influenced by the specific plant exudates and bee secretions incorporated during its formation. For instance, its characteristic odor can vary, exhibiting notes such as clove, eucalyptus, cinnamon, or pine, depending on the predominant plant sources utilized by the bees [35]. Beyond olfactory distinctions, the color of propolis can range from yellow to dark brown according to the trees, shrubs, and sap from which the resin was derived [9,10,11,12,13,14] (Figure 1), while its consistency can vary from a hard and brittle state at low temperatures to a soft, sticky, and rubbery texture at higher temperatures, with these attributes being directly tied to the specific plant resins incorporated. This material typically presents as granules of varying sizes and hues, and it transforms from a hard, crumbly texture to a viscous and sticky substance upon slight heating, generally melting at approximately 70 °C [28]. Its density, ranging from 1.11 to 1.27 g/cm^3^, is primarily influenced by its wax content. However, it is crucial to acknowledge that reported density values, such as 1.186, should be interpreted with caution due to the pronounced variability in propolis composition, particularly wax content, which can substantially influence this physical parameter [7].

## 3. Chemical Composition of Propolis

The chemical composition of propolis is highly variable and is influenced by its botanical source, geographical origin, and seasonal conditions. Typically, it contains 50–60% plant resins and balsams rich in phenolic compounds and flavonoids, 25–35% beeswax, 5–10% essential and aromatic oils, and 5–10% pollen, in addition to minor constituents such as sugars, fatty acids, steroids, terpenoids, vitamins, minerals, and trace elements [36]. This diverse chemical profile is largely responsible for the broad spectrum of biological activities associated with propolis, including antioxidant, antimicrobial, anti-inflammatory, and immunomodulatory properties [11]. Geographical origin plays a crucial role in determining the composition of propolis, leading to significant chemical heterogeneity that presents challenges for standardization and commercialization. Among the various types, poplar propolis is the most extensively investigated and is characterized by high levels of flavonoids and phenylpropanoids, whereas tropical green and red propolis are particularly enriched in coumaric acids and isoflavonoids. The major constituents of poplar propolis include flavonoids, volatile compounds, hydrocarbons, steroids, and vitamins [37,38,39,40].

The chemical structure of propolis is crucial for understanding its biological properties [41,42]. The chemical structure of propolis varies greatly, influenced by the variety of plant species growing near the hive, as well as its geographic location [2,17,43].

Table 1 presents the main chemical classes of propolis and their representative compounds. Propolis obtained from Europe is high in phenolic compounds, flavonoids, and essential oils, whereas that obtained from Australia and South America is rich in diterpenes and triterpenes, as well as phenolic compounds [40]. Another study stated that the chemical profiles of poplar-type propolis collected from different countries, such as Korea, China, Taiwan, New Zealand, and European countries, are similar [44]. In contrast, the study in [45] investigated the flavonoids, total phenols, and total antioxidant capabilities, as well as the phenolic profile and bioaccessibility, of propolis obtained from various regions in Turkey. Their findings indicated that the biological components of the propolis samples were statistically significantly different, implying that these differences could be due to climate and the flora near the hive. The study in [46] identified a diverse range of bioactive compounds in propolis samples collected from geographically distinct locations across Egypt.

## 4. Methods for Obtaining Propolis Extract

Because of its high level of impurities (mostly waxes, resin, and dangerous compounds) and poor water solubility, raw propolis is not acceptable for use in food items, regardless of its origin. As a result, solvent extraction is required to purify propolis. Propolis extracts are obtained by soaking propolis in ethanol and/or water for some time. Ethanol extraction is very beneficial for producing wax-free propolis extracts that are high in bioactive components [56,58]. But ethanol extraction has drawbacks; for example, it produces a strong taste, and certain customers have a sensitivity to alcohol. Data in Table 2. present a comparative overview of propolis extraction techniques: solvents, phenolic yield, and extraction time.

There are currently two types of extraction techniques: traditional extraction methods and contemporary extraction methods. Ultrasound-assisted, supercritical fluid, microwave-assisted, pressurized liquid, and pressurized hot water extractions are examples of current extraction technologies that have been developed as alternatives to traditional methods. The traditional technique of solid–liquid extraction involves the use of a solvent and a leaching process that includes maceration, percolation, and Soxhlet extraction [56].

Conventional extraction procedures for propolis are associated with high solvent consumption, lengthy processing times, and limited selectivity. These limitations have driven the development of more efficient and environmentally sustainable extraction technologies capable of maximizing bioactive compound recovery.

Data on the manufacturing of propolis aqueous solutions are limited; nonetheless, their undeniable benefits are their low cost and lack of alcohol in their structure. Water extraction procedures have the following disadvantages: the propolis extract obtained has a strong flavor and scent, as well as a lower phenolic content than that obtained through alcoholic extraction because of the poor water solubility [58]. A non-ethanolic solvent combination based on polyethylene glycol is an alternative to the aforementioned solvents that allows for the extraction of more active components from propolis than extractions using water [49]. A previous investigation [52] compared Brazilian propolis extract obtained using the traditional ethanol extraction method with that obtained using the supercritical CO_2_ extraction method. In comparison to the propolis extract obtained using standard ethanol extraction, the authors discovered a greater concentration of active components in the propolis extract obtained using the supercritical CO_2_ extraction method. Supercritical CO_2_ extracts these target compounds with greater selectivity, and they were found to be particularly correlated with the antioxidant and antimicrobial properties of the propolis extract.

The microwave-assisted extraction method is one of the extraction technologies that could significantly reduce the time required for propolis extraction by allowing uniform energy distribution in the solid matrix and solvents [47]. Microwaves use large amounts of energy in the form of heat for extraction. Moisture in the solid matrix absorbs microwave energy, causing internal overheating and promoting digestion of the solid, thereby improving the recovery of the main bioactive component in a short interval. Compared to traditional extraction methods such as maceration, propolis extraction through MAE involves less solvent degradation and no serious compound degradation, and it can be performed in less time [48]. Microwave-assisted extraction (MAE) is the fastest overall, achieving 73% extract yield in just 2 × 10 s of irradiation, compared to 58% from traditional maceration after 72 h. Ultrasonication extraction has also been presented as a fast and efficient extraction method for propolis [50]. Acoustic cavitation with ultrasonic energy in the sample enables rapid extraction of the main bioactive compounds. This technique has shown strong potential in reducing the time of extraction and increasing the yield. Ultrasonication extraction achieved a higher proportion of phenolic compounds in extracts than microwave-assisted extraction. Ultrasound extraction resulted in a phenolic content of 50% in 30 min, while microwave-assisted extraction resulted in a 40% phenolic content after 2 × 10 s of microwave irradiation of the sample. In addition, this strategy saved time and labor [59].

The use of nanofiltration in membrane concentration processes, or especially in propolis extraction, is increasing due to several advantages, such as low energy consumption, no phase transitions, and low-temperature operation. This method depends on the principle of selective penetration of dissolved substances through semi-permeable membranes, inorganic membranes, or polymer membranes. Most processes, such as reverse osmosis, nanofiltration, ultrafiltration, and microfiltration, use mechanical pressure to drive the transport of material through the membrane. The nanofiltration process shows up to 90% efficiency in the extraction of phenol and flavonoid compounds in aqueous and alcoholic solutions [60]. Through the nanofiltration method, 72 mg/g of flavonoid compounds and 105 mg/g of phenolic compounds were obtained in the ethanol solution of propolis. On the other hand, through the same procedure, 97 mg/g of flavonoid compounds and 104.7 mg/g of phenolic compounds were obtained in the water solution of propolis [60,61].

Pressurized liquid extraction is becoming an important technique for applications in extraction processes, including the analysis of food and propolis extraction processes. It requires less solvent, is environmentally friendly, enables efficient analytical analysis in an inert and closed environment, and enables high-temperature extraction. Key extraction parameters like pressure, solvent type, temperature, time of extraction, and cell size have been identified [53]. A phenolic content recovery rate of 95–98% can be achieved using the pressurized liquid extraction method.

Thermal pressure water technology is another extraction technique that has been used to extract the main molecules from propolis. This method is based on water and its dielectric properties, viscosity, and surface tension, which are similar to those of organic solvents, and, at the same time, it has many advantages over conventional extraction with organic solvents. A quantitative comparison [62] demonstrated that the contents of seven flavonoids, caffeic acid phenol esters, and four phenolic compounds obtained from thermal pressure water extraction were 35% higher than those obtained from hot water extraction at atmospheric pressure. Furthermore, the addition of natural detergents to the process increased the extraction of the specified compound by 44% in comparison to the process without natural detergents [62].

**Table 2:** A comparative overview of propolis extraction techniques: solvents, phenolic yield, and extraction time.

Extraction Method	Solvent/Medium	Key Advantages	Key Disadvantages	Phenol Yield/Efficiency *	Time	References
Ethanolic (maceration)	Ethanol	Wax-free extract; high bioactive content	Strong taste; alcohol sensitivity; large solvent volume; time-consuming	~58% extract yield (after 72 h)	Very long (up to 72 h)	[52,59,60]
Polyethylene glycol (PEG)	Non-ethanolic PEG	Better active component extraction than water alone	Limited data; not widely studied	Higher than water	Moderate	[61,63]
Supercritical CO_2_	CO_2_ (supercritical)	Higher active component concentration; greater selectivity; superior antioxidant/antimicrobial properties	Complex equipment; high cost	Higher than ethanol	Moderate	[53]
Microwave-Assisted (MAE)	Ethanol/solvent	Fast; uniform energy distribution; less solvent degradation; no serious compound degradation	High energy consumption	~73% extract yield	Very short (2 × 10 s irradiation)	[62,64]
Ultrasonication (UAE)	Ethanol/solvent	Fast; higher phenol yield than MAE; saves time and labor	Equipment required	50% phenolic content in 30 min	Short (30 min)	[65,66]
Nanofiltration	Ethanol or water	Low energy; no phase transitions; low temperature; up to 90% efficiency	Membrane cost and maintenance	Flavonoid: 72–97 mg/g; Phenol: 104.7–105 mg/g	Moderate	[67,68]
Pressurized Liquid Extraction (PLE)	Solvent (various)	Less solvent; environmentally friendly; closed/inert environment; high-temp extraction	Pressure equipment needed	95–98% phenol recovery	Short–moderate	[69]
Thermal Pressurized Water (TPWE)	Hot pressurized water	No organic solvents; eco-friendly; water acts like organic solvent	Specialized equipment; limited data	35–44% higher than atmospheric hot water	Moderate	[70]

* Yield and efficiency values are approximate and vary depending on propolis origin, solvent concentration, and extraction parameters. All values are drawn from primary studies cited per row.

## 5. Analytical Techniques for Propolis Quality Control and Authentication

The quality control and authentication of propolis for food applications require robust analytical techniques capable of addressing its complex and variable chemical composition. High-performance liquid chromatography coupled with mass spectrometry (LC-MS/MS) is widely employed for the targeted quantification of key bioactive markers, including galangin, chrysin, naringenin, caffeic acid phenethyl ester (CAPE), and artepillin C, which serve as important quality indicators [71,72,73]. Complementary gas chromatography–mass spectrometry (GC-MS) is essential for profiling volatile and semi-volatile compounds, such as terpenes, fatty acids, steroids, and aromatic aldehydes, which contribute to the sensory and functional properties of propolis and support chemotaxonomic classification [57,65]. Nuclear magnetic resonance (NMR) spectroscopy, including metabolomics and quantitative NMR approaches, provides comprehensive compositional characterization and structural elucidation of propolis constituents while enabling quantification without the need for reference standards [66]. Rapid, non-destructive screening methods such as attenuated total reflectance Fourier-transform infrared spectroscopy (ATR-FTIR) and near-infrared (NIR) spectroscopy, combined with chemometric analysis, have demonstrated high accuracy for geographic traceability, adulteration detection, and estimation of phenolic and flavonoid contents [72,73]. In addition, inductively coupled plasma mass spectrometry (ICP-MS) is used to monitor elemental composition and detect heavy metal contaminants, ensuring compliance with food safety requirements [71]. Collectively, the integration of spectroscopic screening, chromatographic quantification, NMR characterization, and elemental profiling provides a comprehensive and reliable framework for propolis authentication, quality assurance, and the correlation of chemical composition with biological functionality. A summary of the main analytical techniques used for the chemical characterization, authentication, and quality control of propolis, together with their major applications and analytical targets, is presented in Table 3.

## 6. Bioactive Compounds: Activity of Propolis

The biological activity of propolis is intrinsically linked to its diverse and chemically complex phytochemical composition. Propolis exerts its main functional effects through three well-documented mechanisms: antioxidant activity, whereby phenolic compounds and flavonoids neutralize reactive oxygen species and inhibit lipid peroxidation; antimicrobial activity, in which flavonoids and phenolic acids disrupt bacterial cell membrane integrity and inhibit microbial enzyme systems; and anti-inflammatory activity, mediated through the suppression of pro-inflammatory cytokines and the inhibition of cyclooxygenase and lipoxygenase pathways. Beyond these core bioactivities, propolis has also demonstrated antifungal, antiviral, and immunomodulatory properties, further broadening its functional relevance. Collectively, these activities underpin the growing scientific and industrial interest in propolis as a multifunctional natural bioactive ingredient, particularly for applications in food preservation and functional food development, where its capacity to simultaneously retard oxidative deterioration and inhibit microbial spoilage represents a significant technological advantage over synthetic additives.

The preceding sections have established the chemical diversity of propolis and the analytical platforms used to characterize it. The following section critically evaluates how these chemical constituents translate into measurable biological activities relevant to food preservation applications, with particular emphasis on the evidence base, its limitations, and its food technology implications.

### 6.1. Antioxidant Properties of Propolis

Among natural antioxidants, propolis is particularly noteworthy for its capacity to prevent lipid oxidation in foods, owing to the synergistic action of its diverse phytochemicals, with flavonoids and phenolic acids representing its primary active constituents [69]. The complex chemical profile enables propolis to mitigate oxidative stress through free radical scavenging and the modulation of antioxidant enzyme activities [70]. These mechanisms collectively protect against reactive oxygen species-mediated cellular damage, including lipid peroxidation and DNA degradation, processes central to the pathogenesis of numerous chronic diseases [71]. Furthermore, the intricate interplay of these bioactive compounds within propolis contributes to its multifaceted antioxidative mechanisms, extending beyond direct free radical neutralization to encompass metal chelation and the inhibition of pro-oxidant enzymes. Specifically, its bioflavonoids play a critical role in scavenging free radicals, while other components can chelate heavy metals, thereby disrupting their capacity to generate further oxidative stress [55,57]. This multifaceted action underscores the therapeutic potential of propolis in various oxidative stress-related pathologies, from inflammation to cellular damage [55]. Beyond these primary mechanisms, propolis also demonstrates protective effects against liver and kidney damage induced by toxins, further highlighting its comprehensive antioxidative utility [72].

The antioxidant capacity of propolis has been demonstrated through various in vitro assays such as DPPH, ABTS, and FRAP [73]. In the DPPH assay, propolis polyphenols donate electrons to the stable radical, reducing its absorbance at 517 nm in a concentration-dependent manner that directly quantifies scavenging efficiency [74]. The strong antioxidant potential observed in propolis across various studies is frequently attributed to its rich content of phenolic compounds and other secondary metabolites [75]. These compounds contribute to the inactivation of oxidation reactions, thereby preventing the formation of deleterious radical species within biological systems [51]. Specifically, propolis exhibits notable DPPH radical scavenging activity, ranging from approximately 20 to 190 μg/mL, depending on its botanical origin and collection season. Although Brazilian green propolis frequently exhibits stronger antioxidant activity than European poplar-type propolis, direct comparison among studies remains difficult because antioxidant assays, extraction solvents, and expression of results vary considerably. Consequently, differences attributed to geographic origin may partially reflect methodological variability rather than intrinsic chemical composition alone. Brazilian green propolis rich in artepillin C typically achieves IC_50_ values of 20–40 μg/mL, while European poplar-type propolis generally shows IC_50_ values of 50–150 μg/mL. FRAP values of 1.2–8.4 mmol Fe^2+^/g extract have been reported for Turkish propolis samples [48], and ABTS radical scavenging activity of 50–85% inhibition at 100 μg/mL has been documented for ethanolic extracts of European propolis. These values should always be interpreted in the context of the extraction solvent, propolis: solvent ratio, and assay conditions, as these factors substantially influence the reported antioxidant capacity [76]. For instance, specific propolis samples have demonstrated over 69% DPPH radical scavenging activity at concentrations of 100 ppm, with fractionated propolis exhibiting even higher efficacy [77]. Such variations underscore the importance of geographical origin and extraction methodologies in influencing the antioxidant profile of propolis [78]. This potent radical scavenging ability is predominantly linked to the hydrogen-donating potential of its polyphenolic constituents [7], which actively participate in the stabilization of free radicals [79]. The DPPH assay offers several advantages, including its ease of use, high sensitivity, and suitability for high-throughput screening of numerous samples [80,81]. It should be noted, however, that DPPH and ABTS assays are single-electron transfer-based in vitro methods and do not fully recapitulate the complexity of antioxidant mechanisms in vivo. Therefore, in vitro antioxidant data should be interpreted in conjunction with cellular and in vivo evidence to draw meaningful conclusions regarding the health-relevant antioxidant potential of propolis.

### 6.2. Antimicrobial Activity of Propolis

Propolis has demonstrated broad-spectrum antimicrobial efficacy against a wide range of foodborne pathogens of major public health and food safety significance, including *Listeria monocytogenes*; *Staphylococcus aureus*; methicillin-resistant *S. aureus*; *Salmonella* spp.; *Escherichia coli* O157:H7; *Bacillus* spp.; and fungal contaminants such as *Aspergillus* spp., *Penicillium expansum*, and *Candida* spp. [82,83]. The antimicrobial activity of propolis is primarily attributed to its rich content of polyphenols and flavonoids, whose synergistic interactions disrupt critical cellular structures and metabolic processes in microbial cells, thereby inhibiting proliferation and reducing contamination in food systems [62,84].

Ethanolic propolis extract (EEP) has been shown to inhibit *S. aureus* and MRSA effectively, with minimum inhibitory concentration (MIC) values confirming its potential as a natural preservative in fermented foods where these pathogens pose a significant spoilage and safety risk. The MIC against *S. aureus* is 0.16–1.25 mg/mL, that against MRSA is 0.31–2.5 mg/mL, that against *Listeria monocytogenes* is 0.05–0.4 mg/mL, that against *Candida* spp. is 0.5–4.0 mg/mL, and that against Gram-negative foodborne pathogens such as *E. coli* O157:H7 and *Salmonella* spp. is 2.5–10 mg/mL, reflecting the structural selectivity of propolis polyphenols for Gram-positive over Gram-negative organisms. *Listeria monocytogenes* is a critical cold-chain pathogen of particular concern in dairy and ready-to-eat products, and propolis incorporated into milk demonstrated strong anti-listerial activity during refrigerated storage, effectively suppressing listerial growth under both optimal and suboptimal refrigeration conditions. Collectively, the evidence presented above confirms that propolis extracts exert broad-spectrum antimicrobial activity across diverse food matrices, with efficacy dependent on the concentration, botanical origin, and target microorganism. These effects are primarily mediated through the direct interaction of propolis bioactive constituents with microbial cell structures, as elaborated in the mechanistic discussion above.

Having established the biological activity profile of propolis and its main mechanisms of action, Section 7 addresses the practical technological strategies developed to overcome the physicochemical barriers that currently limit the direct incorporation of propolis into food systems. These strategies must be understood in direct relation to the specific biological activities described above, as their ultimate objective is to deliver functionally active propolis at concentrations sufficient to achieve meaningful antioxidant and antimicrobial effects within the constraints of sensory acceptability and food matrix compatibility.

## 7. Strategies for Using Propolis in Food Technology

As previously discussed, propolis has garnered significant interest in food science due to its potent antimicrobial and antioxidant properties, attributable to its rich composition of bioactive compounds [27]. These diverse biological activities position propolis as an ideal choice for integration into various food systems, serving as a functional ingredient to enhance both preservation and nutritional profiles [85]. Its application is particularly appealing given the growing consumer demand for natural food additives over synthetic alternatives, addressing concerns about food safety and health [86,87]. However, the widespread implementation of propolis in food technology faces challenges, primarily due to its unpalatable bitter taste and flavor, which can negatively impact the sensory attributes of food products [54]. This limitation, coupled with its low water solubility, restricts its direct incorporation into many food matrices [85].

The main strategies for incorporating propolis into food systems can be categorized as follows:

1. Direct addition of propolis to food products: Propolis can be added directly to food products, such as honey, yogurt, milk, and chocolate. This is a simple and effective way to increase the nutritional value and health benefits of food products.
2. Encapsulation of propolis: Encapsulation is a process of trapping propolis in a protective coating. This can be performed using a variety of materials, such as alginates, chitosan, and liposomes. Encapsulation protects propolis from degradation and improves its bioavailability.
3. Use of propolis extracts: Propolis extracts can be obtained using a variety of solvents, such as ethanol, water, and vegetable oils. Propolis extracts are applied to foods directly, or products are immersed in them; additionally, foods can be covered with coatings containing propolis extracts. Both strategies can be used to minimize and remove pathogens present in foods, as well as saprophytic bacteria in foods. Because of their antioxidative properties, propolis extracts have been used to protect fruit and fruit juices from oxidation during storage [88].
4. Fermentation of propolis: Fermentation of propolis using lactic acid bacteria can improve its flavor and increase its content of bioactive compounds. Fermented propolis can be used to develop functional food products, such as probiotic yogurt and cheese.
5. Combination of propolis with other functional ingredients: Propolis can be combined with other functional ingredients, such as vitamins, minerals, and prebiotics, to achieve synergistic health benefits.

**Figure 2:**
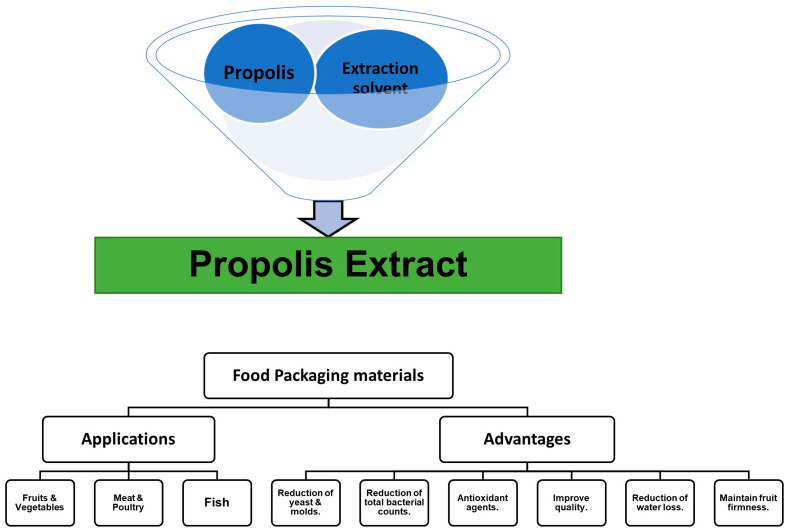
Applications of propolis extract in food packaging. Source: developed by the authors.

The use of propolis in food packaging might provide an alternative method to its direct application in food, which is currently prohibited because of its strong and unique smell, which could affect food sensory characteristics [89]. Inclusion of propolis into films or coatings makes it easier to apply its antibacterial qualities to food products. Biopolymer coatings mixed with propolis extracts can have a synergistic impact on the prevention of fungal infections in vegetables and fruits [90]. Figure 2 shows the main advantages of using propolis in food packaging materials.

Gum arabic coating containing 5% propolis extract and 0.1% cinnamon oil provided better protection against the development of *C. capsica* in peppers than coatings with gum arabic or gum arabic with cinnamon oil only [91]. Similarly, hydroxypropyl methylcellulose coating material with 1.5% Spanish propolis extract had a greater inhibitory effect on aerobic bacteria counts, yeasts, and molds than pure hydroxypropyl methylcellulose [92].

The widespread industrial adoption of propolis in food products remains fundamentally constrained by two main physicochemical limitations: its characteristic strong odor and its poor water solubility [80,93]. The volatile terpenes, aromatic aldehydes, and phenolic esters responsible for propolis’s intense aroma reduce sensory acceptance at biologically effective concentrations, while its hydrophobic nature prevents uniform dispersion in aqueous food matrices, collectively restricting both bioavailability and antimicrobial efficacy [59,80,93]. To overcome these barriers, current research has converged on three main technological strategies. Microencapsulation, using wall materials such as alginate, chitosan, whey protein, or gum arabic, has demonstrated efficacy in masking propolis’s flavor while protecting bioactive compounds from oxidative degradation and enabling controlled release within the food matrix [94]. Additionally, the development of water-soluble propolis extracts through polyethylene glycol-based solvents, cyclodextrin complexation, or membrane filtration provides a direct strategy for integrating propolis into aqueous matrices without compromising the phenolic content or biological activity [61,67,68]. Addressing these limitations through continued innovation in delivery and formulation technologies, therefore, represents an essential condition for the broader commercialization of propolis as a natural food preservative. Table 4 presents some recent studies related to the addition of propolis and its extract to food products.

The formulation strategies outlined in Section 7 provide the technological foundation for incorporating propolis into diverse food systems. Section 8 synthesizes experimental evidence on the performance of propolis and its formulations across specific food matrices, critically evaluating efficacy, practical feasibility, and the sensory and safety constraints that modulate real-world applicability.

## 8. Effect of Propolis Incorporation on Different Food Matrices

The intricate relationship between the sensory attributes of food products and their ultimate acceptance by consumers is a critical determinant in the efficacy of dietary interventions aimed at mitigating chronic disease prevalence [103]. This paradigm underscores the necessity of integrating consumer-centric sensory evaluation into the development of functional foods, as favorable organoleptic properties are paramount for sustained dietary adherence and, consequently, for realizing long-term health benefits [104]. Despite the demonstrated health potential of various foods, their actual beneficial impact hinges on consumer availability, choice, preference, and consistent consumption [105]. The burgeoning interest in self-administered healthcare among consumers has propelled the demand for functional foods, yet their successful integration into daily diets depends heavily on aligning scientific advancements with consumer perceptions and expectations [106]. This necessitates a comprehensive understanding of consumer behavior, encompassing factors such as perceived health benefits, willingness to pay, and, crucially, sensory appeal, which collectively influence the adoption and sustained consumption of health-promoting food products [106,107]. Specifically, the sensory characteristics encompassing appearance, aroma, taste, mouthfeel, and texture directly modulate consumer responses, influencing initial acceptance, sustained consumption patterns, and overall dietary compliance. Furthermore, consumers with pre-existing health conditions, such as diabetes or metabolic syndrome, often exhibit altered sensory perceptions, which can significantly influence their food choices and dietary compliance [63]. Therefore, understanding and actively managing the sensory profiles of food products are crucial for promoting diet-related disease prevention and overall health, particularly given the challenges associated with reformulating processed foods to enhance nutritional value without compromising sensory appeal [103]. However, developing novel functional foods, though time-consuming and expensive, must prioritize mitigating skepticism and uncertainty among consumers to ensure their successful uptake [95].

### 8.1. Dairy Products

Several studies have highlighted the influence of propolis extracts on dairy products (yogurt, milk, and cheese). In general, propolis extract in raw milk resulted in considerably higher acidity values, which might be related to improved lactic acid bacteria action, resulting in increased sugar breakdown in the milk and an increase in acidity [97]. Furthermore, propolis extract significantly increased phenolic and flavonoid contents, as well as antioxidant capacity, compared to control yogurt [42]. The increase in phenolic content and antioxidant capability can be attributed to the fact that propolis extracts exhibit strong antioxidant activity and produce a high amount when added to yogurt and milk.

Propolis-enriched dairy products exhibit improved antioxidant properties while retaining desirable sensory characteristics, making them appealing to health-conscious consumers [99]. However, the challenge lies in balancing the concentration of propolis to ensure that its strong flavor does not overpower the product’s original taste, highlighting the importance of careful formulation and consumer testing [103]. This balance is crucial, as successful integration of propolis can lead to innovative products that meet the growing demand for functional foods without sacrificing sensory enjoyment.

The addition of propolis extract also caused color changes in the product, according to the authors. Propolis extract, in combination with conjugated linoleic acid, was studied as an antioxidant in dairy beverages. The main benefit of employing propolis extract was its increased antioxidant capacity and ability to decrease off-flavors related to aldehyde formation, as no nutritional loss was seen during the manufacturing and storage of dairy beverages.

### 8.2. Meat and Fish Products

In fish products, application of ethanolic propolis extract to sardine fillets resulted in lower lipid oxidation and reduced bacterial growth compared to untreated controls. These effects are predominantly attributed to the direct interaction of propolis bioactive components with bacterial cell structures, disrupting membrane integrity and inhibiting key enzymatic pathways essential for microbial survival [108].

In comparison to the control, adding 5% spray-dried propolis to an appropriate fish burger resulted in about a 3 times higher phenolic content and 4 times higher antioxidant activity [98]. The addition of microencapsulated propolis extract (0.3 g/kg meat) to burger meat had a stronger inhibitory effect on lipid oxidation in the burger meat than sodium erythrobate, a synthetic antioxidant [94]. Additionally, adding propolis extract (0.05–0.1%) to Italian salamis inhibited oxidative rancidity and could be applied as a natural antioxidant in food products [96].

### 8.3. Fruits and Juices

Moreover, the successful integration of propolis into food products hinges not only on its functional benefits but also on the careful consideration of the extraction methods used, as these can significantly influence the bioactive compounds’ stability and sensory properties. For instance, advanced extraction techniques like supercritical fluid-assisted extraction have been shown to enhance both the yield and preservation of propolis’s beneficial components, thereby ensuring that the final product remains appealing to consumers while maximizing health benefits [27]. Additionally, the incorporation of propolis into everyday foods such as dairy products and baked goods has demonstrated the potential to improve their nutritional profiles without compromising taste, thereby addressing the growing consumer demand for healthier options [103]. This balance between functionality and sensory quality is vital, as it directly impacts consumer acceptance and the overall success of these innovative food products in the market. The challenge lies in achieving this balance while navigating regulatory approvals and potential allergenicity concerns associated with propolis use in food applications. Therefore, ongoing research and development are essential to optimize propolis formulations and ensure that they meet safety standards while appealing to health-conscious consumers. When propolis extract was mixed with ascorbic acid in soft orange beverages, researchers discovered a synergic effect on antioxidant activity.

Propolis has also proven highly effective against fungal contaminants responsible for spoilage and mycotoxin production in food products. The addition of ethanolic propolis extract at 0.2% to apple juice successfully suppressed the growth of patulin-producing Penicillium expansum strains, a particularly significant finding given that patulin is a regulated mycotoxin with established maximum limits in fruit-based products [100]. Turkish ethanolic propolis extract added to non-pasteurized fruit juices at concentrations of 0.01 to 0.4 mg/mL fully suppressed yeast growth, with antibacterial properties exceeding those of sodium benzoate, a widely used synthetic preservative. Similarly, propolis extract suppressed yeast and mold growth in freshly squeezed pomegranate juice for 23 days [101], and gum arabic coating containing 5% propolis extract and 0.1% cinnamon oil provided superior protection against Colletotrichum capsaicin peppers than a coating with gum arabic alone.

Propolis applications have been observed to minimize the weight loss of numerous foods under storage conditions, including cherry, papaya, mango, bananas, orange, and cucumber. This decrease in weight could be attributed to propolis’s properties that limit transpiration and respiration. Another study indicated that ethanol extract of propolis at a concentration of 1.0% protected pomegranate fruit weight under storage at 6.5 1 °C and 90–95% humidity levels [82]. The weight losses of fruits pretreated with propolis and those not treated were 11.3% and 19.8%, respectively, after storage for 5 months [109].

The incorporation of propolis extracts not only enhances the barrier properties of packaging but also imbues it with antimicrobial and antioxidant characteristics, which can further extend the shelf life of fresh produce. This dual functionality is particularly beneficial in the context of modified atmosphere packaging (MAP), where the preservation of quality is paramount for fruits with high respiration rates [83]. The use of active packaging films that leverage the bioactive compounds in propolis has shown promise in maintaining the sensory and nutritional quality of fruits during storage, as it mitigates the growth of spoilage microorganisms while reducing oxidative stress [90]. Such advancements underscore the potential for biopolymers infused with natural extracts to revolutionize food preservation techniques, aligning with the growing consumer demand for sustainable and health-conscious packaging solutions.

### 8.4. Packaging Applications

The potential for biopolymers and natural extracts like propolis to enhance food packaging aligns with a broader movement towards a circular economy, where the focus is on sustainability not just in materials but also in the entire lifecycle of products. This approach encourages not only the use of renewable resources but also the design of packaging that can be easily recycled or composted after use, thus minimizing waste and promoting resource efficiency. As companies increasingly recognize the strategic importance of sustainability in their business models, they are investing in research and development to overcome existing barriers to the widespread adoption of these materials, such as cost and scalability challenges [84]. Additionally, the integration of consumer education initiatives can play a vital role in fostering acceptance and understanding of these innovative packaging solutions, ultimately leading to more informed purchasing decisions and a stronger market for sustainable products [110]. This holistic perspective on packaging not only addresses immediate environmental concerns but also positions brands as leaders in the transition towards a more sustainable future, resonating with the values of today’s eco-conscious consumers.

Furthermore, the integration of propolis-based active packaging films aligns with emerging trends in the food industry, where there is an increasing shift towards natural and organic products. As consumers become more discerning about the ingredients in their food, the demand for packaging that not only preserves quality but also contributes to health and wellness is on the rise. For instance, the use of biopolymers combined with propolis not only addresses sustainability concerns but also taps into the growing market for functional foods, which are designed to offer health benefits beyond basic nutrition [111]. Additionally, the potential for these innovative packaging solutions to reduce food waste and the escalating global issue highlights their importance in fostering a more sustainable food system. By extending the shelf life of perishable items, such solutions could significantly curb the environmental impact associated with food spoilage and disposal, thereby reinforcing the role of packaging innovations in achieving broader sustainability goals.

Due to their strong hydrophobicity, propolis extracts can form a biodegradable barrier on fruit surfaces, preventing the flow of water and gases through the food surface. This mitigates post-harvest weight loss and deterioration, thereby extending the shelf life of fresh produce. Furthermore, propolis-based coatings actively reduce microbial proliferation, a significant factor in post-harvest spoilage, through direct antimicrobial action attributed to compounds such as cinnamic, ferulic, and caffeic acids [108]. Beyond mere barrier formation, the inherent antimicrobial properties of propolis extracts offer a sophisticated mechanism for maintaining fruit quality and extending freshness [112]. This makes propolis extracts a promising natural alternative to synthetic fungicides, which often raise concerns regarding food safety and environmental impact [113].

### 8.5. Challenges and Limitations for Food Applications

The chemical composition of propolis is highly dependent on the botanical source, bee species, season, and geographic location. Consequently, two propolis samples collected from different regions may exhibit substantially different concentrations of flavonoids, phenolic acids, and terpenoids, resulting in inconsistent biological activity.

The significant variability in its chemical composition and the potential for allergenic reactions present substantial obstacles to achieving standardized quality control in commercial food preservation [87]. The reliance on distinct botanical sources and environmental conditions leads to fluctuating bioactive profiles, which complicates the establishment of universal safety and efficacy benchmarks [27]. Specifically, the high concentration of volatile phenolic acids often imparts a persistent bitterness, increased viscosity, and astringency, which often affect consumer acceptability [114]. To address these organoleptic drawbacks, advanced techniques such as spray-drying and microencapsulation are increasingly employed to mask off-flavors and improve solubility, although determining the optimal concentration—typically limited to low ranges—remains critical to preserving both sensory quality and functional integrity [115].

## 9. Future of Propolis in Food Technology

One of the most pressing challenges facing the application of propolis in food is the absence of universally accepted standardization criteria and quality control frameworks specific to propolis intended for human consumption. The chemical composition of propolis varies, making it inherently difficult to define uniform quality specifications applicable across all propolis types and origins. Future research should prioritize the development of validated, type-specific quality standards that define minimum thresholds for key bioactive compounds such as total flavonoid and phenolic contents, CAPE concentration, and artepillin C levels, alongside the maximum permissible limits for contaminants including heavy metals, pesticide residues, and wax content. The establishment of such frameworks, in collaboration with international regulatory authorities, including EFSA, the FDA, and the Codex Alimentarius Commission, would provide the regulatory foundation necessary for the broader commercialization of propolis-based food products in global markets.

The integration of propolis into commercial food products aligns with converging market forces: escalating consumer demand for natural, minimally processed foods; growing preference for transparently sourced, sustainable ingredients; and expanding evidence base confirming propolis’s functional efficacy. Sourcing propolis from apiaries practicing sustainable beekeeping further reinforces the health and environmental narratives sought by contemporary consumers while simultaneously supporting biodiversity and pollinator conservation [99]. Moreover, the exploration of innovative extraction methods and formulations may lead to more effective ways to harness the benefits of propolis, addressing challenges such as variability in its chemical composition due to environmental factors [87]. As the demand for natural and functional foods rises, the potential for propolis to become a staple ingredient in health-conscious markets appears promising, paving the way for further technological advancements and regulatory frameworks that could facilitate its broader application in the food industry.

The potential for propolis to enhance not only the nutritional value but also the sensory attributes of food products cannot be overlooked. Recent studies indicate that propolis can positively influence flavor profiles, making it an attractive ingredient for food developers aiming to satisfy the palates of health-conscious consumers while maintaining product appeal [116]. This dual capability could lead to innovative culinary applications, such as in gourmet health foods or functional beverages, where taste and health benefits are paramount. As food technology progresses, the exploration of propolis’s role in flavor enhancement could open new avenues for product differentiation in a competitive market, reinforcing its status as a versatile natural additive. Furthermore, the integration of propolis into mainstream food products may also stimulate interest in other bee-derived ingredients, promoting a holistic approach to utilizing the full spectrum of benefits provided by apiculture, thus fostering a deeper connection between consumers and sustainable agricultural practices.

As the food industry explores the multifaceted applications of propolis, it is also crucial to consider the broader implications of consumer behavior and market trends that influence product acceptance. The rising consumer interest in transparency and sustainability, as evidenced by the shift towards Community-Supported Agriculture (CSA) and local food networks, suggests that consumers are increasingly seeking out products that not only deliver health benefits but also support ethical agricultural practices [100]. This trend may encourage food manufacturers to adopt more sustainable sourcing strategies for propolis, thereby fostering a deeper connection between consumers and the origins of their food. Additionally, the integration of propolis into various culinary applications could serve as a gateway for consumers to explore other bee-derived products, enhancing their understanding of the ecological importance of bees and the necessity of protecting these vital pollinators. As this awareness grows, it could drive demand for innovative products that celebrate the diverse benefits of apiculture, ultimately contributing to a more resilient and sustainable food system.

Given these challenges, research has increasingly focused on incorporating propolis into edible films or surface applications for food products, which can circumvent issues related to taste while still leveraging its preservative qualities [54]. These applications often exploit propolis’s ability to inhibit lipid oxidation and microbial growth, thereby enhancing the safety and extending the shelf life of perishable food items [101]. This approach is driven by the understanding that propolis contains various bioactive compounds responsible for its antioxidant and antibacterial activities [115]. These properties make propolis a valuable natural additive for food preservation, offering a compelling alternative to synthetic compounds [93,102].

One of the key areas of future research on propolis in food technology will focus on developing new and innovative ways to incorporate propolis into food products while preserving its bioactive compounds and bioavailability. For example, researchers are developing new encapsulation technologies that can protect propolis from degradation and improve its delivery to the body. Another area of future research will focus on developing new propolis-based functional food products with specific health benefits. For example, researchers are developing propolis-containing yogurt and cheese products with probiotic benefits, as well as propolis-containing chocolate products with antioxidant and anti-inflammatory benefits. In addition to developing new propolis-based food products, researchers are also exploring the potential use of propolis as a natural food preservative. Propolis is effective against a wide range of foodborne pathogens, including bacteria, viruses, and fungi. This suggests that propolis could be used as a natural alternative to synthetic food preservatives.

From a regulatory standpoint, the current status of propolis as a food ingredient varies considerably across jurisdictions. In the European Union, propolis has not been assigned a specific E-number, and its use in food products is subject to national regulations rather than harmonized EU-wide legislation, meaning that market access differs substantially among member states. In the United States, propolis holds GRAS status for certain applications; however, no specific FDA-approved food additive designation exists for propolis extracts as a class. In China, propolis has been recognized as a new food resource since 2010, enabling its use in functional food applications under regulatory oversight. The Codex Alimentarius Commission has not yet established the maximum residue limits or quality specifications for propolis in food. This regulatory fragmentation constitutes a major structural barrier to global commercialization and underscores the urgent need for international harmonization of propolis quality standards, maximum usage levels, and safety documentation requirements.

Overall, propolis has the potential to play a significant role in the future of food technology. Its unique properties make it ideal for use in developing functional food products that can improve human health and extend the shelf life of food products. More research is needed to fully understand the health benefits of propolis and to develop new and innovative ways to incorporate propolis into food products. Future research should address formulation and delivery challenges through three main technological pathways: microencapsulation, nanoemulsification, and water-soluble extract development, as elaborated in Section 7, while prioritizing the scale-up feasibility, cost-effectiveness, and regulatory acceptance of each approach. Standardization of quality frameworks in collaboration with the EFSA, FDA, and Codex Alimentarius remains the foundational prerequisite for broad commercialization [96,99].

## 10. Conclusions

This review has documented the remarkable chemical diversity of propolis across global chemotype classifications, including poplar-type, Brazilian green, Brazilian red, Mediterranean diterpenic, and stingless bee propolis, and it has demonstrated how this diversity is the primary determinant of biological activity. Advanced analytical platforms, particularly LC-MS/MS, GC-MS, and 1H-NMR metabolomics, have substantially deepened the characterization of propolis composition, enabling geographic authentication and quality control with unprecedented precision. The antioxidant, antimicrobial, anti-inflammatory, and immunomodulatory properties of propolis have been substantiated across multiple food matrices, including meat, dairy products, fruit juices, and baked goods, confirming its scientific credentials as a multifunctional natural food preservative. Collectively, the evidence reviewed here establishes propolis as a bioactive ingredient with genuine potential to reduce reliance on synthetic preservatives and contribute to the development of functional foods aligned with the consumer demand for clean-label, health-promoting products.

Propolis plays multiple evidence-supported roles in food preservation, mainly as an antioxidant, antimicrobial, and antifungal agent, with the additional capacity to retard undesirable physicochemical deterioration in food matrices. Despite a rapidly expanding evidence base, further research is required before propolis can be widely and reproducibly integrated into commercial food products. Compositional variability among propolis samples from different geographic origins constitutes a significant commercial and regulatory challenge, not a minor formulation issue, because it prevents the establishment of universal quality standards and complicates dose–response comparisons across studies. Resolving this challenge through validated, type-specific analytical reference methods and internationally harmonized regulatory frameworks, therefore, remains the highest priority for translating propolis research into broadly applicable food technology.

Notwithstanding this promising scientific foundation, the translation of propolis into commercial food applications is currently constrained by two main physicochemical barriers that remain unresolved. The first is its intense characteristic odor arising from volatile phenolic esters, terpenes, and aromatic aldehydes, which compromises the sensory acceptability of food products when it is incorporated at concentrations required for antimicrobial efficacy. The second is its inherently low aqueous solubility, which prevents uniform dispersion in water-based food matrices, including beverages, dairy products, and fresh-cut produce, thereby restricting both bioavailability and antimicrobial activity in these systems. These are not superficial formulation challenges but fundamental physicochemical constraints that must be technically resolved before propolis can be widely and reproducibly applied as a commercial food preservative.

Three main technological strategies have been investigated to overcome these barriers. Microencapsulation using biopolymer wall materials, including alginate, chitosan, whey protein, and gum arabic, has demonstrated efficacy in masking propolis odor, protecting bioactive compounds from oxidative degradation, and enabling controlled release within food matrices. Nanoemulsification reduces propolis particle size to the nanoscale, enhancing both water dispersibility and bioavailability, with promising results in liquid food systems. The development of water-soluble propolis extracts through polyethylene glycol-based solvents, cyclodextrin complexation, or membrane filtration provides a further approach for integration into aqueous food systems without compromising the phenolic content or biological activity. However, despite proof-of-concept efficacy, none of these strategies have yet achieved the cost-effectiveness, production scalability, or regulatory acceptance required for widespread industrial adoption. The additional challenge of compositional variability between geographic origins, which prevents the establishment of universal quality standards and complicates dose–response comparisons across studies, constitutes a significant commercial and regulatory barrier that must be addressed in parallel with formulation development.

To realize the full potential of propolis as a safe, effective, and consumer-acceptable natural food preservative, future research must address four prioritized gaps. First, the development of validated, type-specific analytical reference methods and internationally harmonized regulatory frameworks is essential to resolve the standardization deficit and enable reproducible quality control across propolis types and geographic origins. Second, in vivo validation of food matrix bioavailability is required to confirm that bioactive compounds retain their efficacy following encapsulation or formulation processing and after passage through the gastrointestinal tract. Third, systematic sensory evaluation at effective preservative concentrations using standardized consumer panels across diverse food matrices is needed to define the precise odor and flavor thresholds that current encapsulation technologies must achieve for commercial acceptance. Fourth, harmonized regulatory frameworks for propolis-derived food additives must be developed across international jurisdictions, including the EFSA, FDA, and Codex Alimentarius, to provide the approval pathways required for market entry. Progress on all four fronts, pursued in an integrated manner, is the necessary condition for propolis to fulfill its considerable and well-documented potential as a functional ingredient in modern food technology.

## Figures and Tables

**Table 1:** Main chemical classes of propolis and their representative compounds.

Chemical Class *	Representative Compounds	Propolis Type/Region	Approx. % Composition	References
Flavonoids	Pinocembrin, chrysin, galangin, quercetin, luteolin, apigenin, kaempferol, naringenin, pinobanksin	Poplar-type (Europe, China, N. America)	~30–40% of resin fraction	[9,11,47,48]
Phenolic acids and esters	Caffeic acid, ferulic acid, p-coumaric acid, cinnamic acid, CAPE, chlorogenic acid	All types; dominant in poplar-type	~15–25%	[33,49,50,51]
Prenylated phenolics	Artepillin C, baccharin, drupanin (prenylated cinnamates)	Brazilian green (*Baccharis dracunculifolia*)	Up to ~50% in green propolis	[52,53,54]
Isoflavonoids and pterocarpans	Medicarpin, isosativan, formononetin, biochanin A, vestitol	Brazilian red, Cuban red (*Dalbergia ecastaphyllum*)	~20–35%	[24,29,55]
Diterpenes	Communic acid, labdane-type diterpenes, ferruginol, totarol, ent-kaurene	Mediterranean (Cupressus, conifers); Pacific; Canary Islands	~10–30%	[10,37,56]
Terpenoids (mono- and sesqui-)	α-pinene, β-caryophyllene, β-eudesmol, limonene, caryophyllene oxide	Volatile fraction; Mediterranean, African	5–10% (volatile fraction)	[34,37,57]
Benzophenones	Guttiferone E, xanthochymol, oblongifolin A, nemorosone	Brazilian brown (*Clusia* spp., *Symphonia*)	~15–30%	[25,30,52]
Fatty acids and wax components	Palmitic acid, stearic acid, oleic acid, linolenic acid	All types; higher in stingless bee propolis	8–35% (wax fraction)	[10,56,58]

* Chemical class and percentage composition ranges are based on the cited primary literature. All data in this table are sourced from the references listed in the References Column of each row; no data are derived from unspecified databases.

**Table 3:** Analytical techniques applied to propolis chemical characterization.

Technique	Analytical Capability	Recent Application in Propolis Research	Key References
LC-MS/MS (triple quadrupole)	Targeted quantitation: phenolic acids, flavonoids, CAPE, artepillin C	Authentication of popular type vs. tropical propolis; quality control workflows	[64,67]
GC-MS	Volatile fraction: terpenoids, fatty acids, aromatic aldehydes; semi-volatile profiling	Yucatan stingless bee propolis; Northern Iraq; Ethiopian propolis	[65]
1H-NMR metabolomics	Fingerprinting of whole extract; rapid; simultaneous multi-class identification; machine learning-compatible	Iranian, Brazilian, Greek/Chinese discrimination; seasonal classification	[68]
ATR-FTIR/NIR spectroscopy	Rapid screening; no sample preparation; geographic origin prediction; adulteration detection	Chinese geographic traceability; authentication screening panels	[67]
ICP-MS	Elemental profile (Ca, Mg, Fe, trace metals); complementary to organic profiling	Iraqi propolis; elemental differentiation between regions	[64]

A comprehensive overview of analytical methodologies applied to propolis characterization, including their capabilities, recent applications, and key references, is available in dedicated reviews [72,73].

**Table 4:** Studies related to the use of propolis as a natural food additive.

Food Matrix	Propolis Type/Extract	Concentration Used	Key Effect	Reference
Beef patties/burgers	Ethanolic extract (EEP)	2%	Reduced lipid oxidation; lowest TBA and conjugated diene values during cold storage	[95]
Burger meat (microencapsulated)	Microencapsulated propolis extract	0.3 g/kg meat	Stronger inhibition of lipid oxidation than sodium erythorbate (synthetic antioxidant)	[94]
Italian salami	Ethanolic extract (EEP)	0.05–0.1%	Inhibited oxidative rancidity; applied as a natural antioxidant	[96]
Fresh oriental sausage	Ethanolic extract (EEP)	0.6%	Significantly slowed lipid oxidation; extended shelf life	[97]
Fish burger	Spray-dried propolis (5%)	5%	~3× higher phenolic content; ~4× higher antioxidant activity vs. control	[98]
Fish fillets (presoaking)	Aqueous propolis solution	0.6%	Extended shelf life during storage at 18 °C; recommended as a natural preservative	[99]
Apple juice	Ethanolic extract (EEP)	0.2%	Suppressed growth of patulin-producing *Penicillium expansum*; best results at 0.2%	[100]
Pomegranate juice (fresh)	Ethanolic extract (EEP)	Not specified	Suppressed yeast and mold growth for 23 days	[101]
Orange juice	Propolis extract emulsion	0.20 mg/mL	Significantly reduced growth of *Bacillus* spores	[102]

## Data Availability

No new data were created or analyzed in this study. Data sharing is not applicable to this article.
